# Eating Speed, Eating Frequency, and Their Relationships with Diet Quality, Adiposity, and Metabolic Syndrome, or Its Components

**DOI:** 10.3390/nu13051687

**Published:** 2021-05-15

**Authors:** Tany E. Garcidueñas-Fimbres, Indira Paz-Graniel, Stephanie K. Nishi, Jordi Salas-Salvadó, Nancy Babio

**Affiliations:** 1 Universitat Rovira i Virgili, Departament de Bioquimica i Biotecnologia, Unitat de Nutrició Humana, 43201 Reus, Spain; tanyelizabeth.garciduenas@estudiants.urv.cat (T.E.G.-F.); indiradelsocorro.paz@urv.cat (I.P.-G.); stephanie.nishi@urv.cat (S.K.N.); 2 Institut d’Investigació Sanitària Pere Virgili (IISPV), 43204 Reus, Spain; 3 CIBER Physiology of Obesity and Nutrition (CIBEROBN), Carlos III Health Institute, 28029 Madrid, Spain

**Keywords:** eating speed, eating rate, eating frequency, adiposity, BMI, eating behaviors, metabolic syndrome, MetS

## Abstract

Excess body weight is a major global health concern, particularly due to its associated increased health risks. Several strategies have been proposed to prevent overweight and obesity onset. In the past decade, it has been suggested that eating speed/rate and eating frequency might be related to obesity. The main aim of this narrative review was to summarize existing evidence regarding the impact of eating speed/rate and eating frequency on adiposity, metabolic syndrome (MetS), or diet quality (DQ). For this purpose, a literature search of observational and interventional trials was conducted between June and September 2020 in PubMed and Web of Sciences databases, without any data filters and no limitations for publication date. Results suggest that children and adults with a faster eating speed/rate may be associated with a higher risk of developing adiposity, MetS or its components. Furthermore, a higher eating frequency could be associated with diet quality improvement, lower adiposity, and lower risk of developing MetS or its components. Further interventional trials are warranted to clarify the mechanism by which these eating behaviors might have a potential impact on health.

## 1. Introduction

Weight disorders, such as overweight (OW) and obesity (OB), are prevalent globally, mainly because of unhealthy diets and sedentary behaviors [1]. In accordance with data from the World Health Organization, global obesity has nearly tripled since 1975 [1]. By 2016, more than 1.9 billion adults were overweight, of these over 650 million were obese and 340 million children and adolescents aged 5 to 19 years were overweight or obese. Meanwhile by 2019, over 38 million children under 5 years-old were overweight or obese [1]. Obesity is defined as a health problem that consists mainly of body fat accumulation, with a multifactorial etiology [2]. It is related to an immense variety of short and long-term consequences, which may affect quality of life [3]. Excess body weight (BW) has been associated with metabolic syndrome (abdominal obesity, elevated fasting plasma glucose, low level of high-density lipoprotein cholesterol (HDL-c), hypertriglyceridemia, and hypertension) [4], a complex interrelated network of metabolic risk factors [5], which increases the risk of diabetes and premature death [6].

Childhood obesity is a risk factor for overweight and obesity in adulthood [7]; therefore, its prevention and early treatment are major concerns for public health. Several strategies have been proposed to prevent overweight and obesity onset, where the vast majority are based on avoiding sedentary behaviors and a western high energy density dietary pattern, rich in refined food, sugary beverage consumption, red and processed meat, and low in vegetables, fruits, whole grains, and legumes [8].

In the last decade, several authors have queried [9,10,11] if certain dietary behaviors, such as eating speed/rate, defined as the time required to eat an amount of food [12,13], and eating frequency, the number of eating occasions per day either reported as a meal or a snack [10,11,14,15,16,17], may be related to obesity, MetS, and dietary quality. It was also suggested that these eating behaviors may have a potential influence in the development of chronic diseases, such as diabetes and cardiovascular disease [18]. For example, in Asian populations, eating speed/rate has been associated with adiposity [19,20], but this has not been shown in Europeans [21]. In Europe, a study conducted in Spanish participants found a higher risk for hypertriglyceridemia in individuals with a fast-eating speed [21]. Moreover, significant associations have been observed between eating frequency and diet frequency, adiposity, or cardiovascular risk [22,23]. In children, the evidence is scarce, but it is suggested that these eating behaviors may play an important role in their health maintenance [24,25]. Along these lines, evidence is quite controversial because a frame of reference for adequate values and universal definitions for these behaviors have not been settled [17]. In view of the impact of eating frequency and eating speed/rate may have on health, the aim of this review was to summarize all the available evidence (with no limitation for publication date) of these eating behaviors in relation to body mass index (BMI), body weight, waist circumference (WC), diet quality, and other MetS components in children, adolescents, and adults.

## 2. Materials and Methods

### 2.1. Search Strategy and Selection Criteria

A literature search was performed in PubMed and Web of Science from June to September 2020 without any data filters, identifying observational and interventional trials examining possible associations between certain eating behaviors (e.g., eating speed/rate and eating frequency), diet quality, cardiometabolic biomarkers and adiposity measures in adults (>19 years) and children (≤19 years).

For this narrative review, the following terms were used to define the exposure: eating speed, eating rate, eating frequency, meal frequency and snack frequency. For outcomes, keywords related to adiposity and cardiometabolic biomarkers were searched, such as glucose, cholesterol, blood pressure, adiposity, body weight, BMI, body mass index, WC, as well as diet quality. Furthermore, the search was complemented with a manual search of the reference lists of included articles.

### 2.2. Inclusion and Exclusion Criteria

To be included, articles had to meet the following inclusion criteria: full-text available, observational study, clinical study, clinical trial, journal article, randomized controlled trial, and involving humans, written in the English or Spanish language. A search was performed in the MEDLINE database (PubMed and Web of Science) until September 2020 and all available articles that met inclusion criteria were included. Exclusion criteria included letters to editors, abstracts only, case reports, registered protocols, and ongoing trials.

### 2.3. Study Selection

Two researchers conducted the article review and selection, with any disagreement being settled by consensus. A specific spreadsheet program (Excel, Microsoft Windows 10 Pro) was used for data collection and summarizing descriptive information and results from the included studies. Relevant data comprised of first author’s surname, year of publication, population, study design, exposure, outcome variables and relevant results. The tables were drafted and classified by study design (cross-sectional, longitudinal, or interventional) and age group (children or adult); their eligibility was widely discussed by consensus.

### 2.4. Exposures

Definitions for eating speed, eating rate, and eating frequency display great variability among studies. Below will describe the definitions used in the present review.

According to the literature, eating speed and eating rate are frequently used as synonyms, being mainly defined, as the time required to eat an amount of food. The most frequent assessment method used was based on self-reporting (subjective), but timers, videotapes, and direct observation were occasionally used to objectively assess eating speed or eating rate.

Eating frequency corresponds to the number of eating occasions per day [12], moreover some authors reported meal and/or snack frequency. Eating frequency is often assessed by self-report, but a few authors evaluated frequency and whether it was considered a meal or snack according to the energy percentage contribution (% energy) or based on the hour (clock time) [13,14,15,16].

### 2.5. Outcomes

In the present review, overweight and obesity in adults was defined as a BMI ≥ 25 kg/m^2^ and a BMI ≥ 30 kg/m^2^, respectively. Meanwhile in children, excess body weight was evaluated with BMI in kg/m^2^ (according to IOTF criteria [26,27,28,29], British Growth reference criteria [14] or Central for Disease Control and Prevention criteria [12]) or percentiles (according to CDC criteria [30,31] or WHO criteria [32]), as well as BMI z-scores [4,10,12,14,17,24,25,27,31]. Abdominal obesity, in adults, was defined by WC cut-offs according to ethnicity. In Asian populations, cut-off points were established by having a waist circumference ≥85 cm in men, and ≥80 cm in women [33,34]. For European populations the cut-off point for abdominal obesity was considered as WC > 102 cm in men and >88 cm in women [33]. In children, only one study [32] evaluated abdominal obesity, which was defined as a waist-hip ratio >0.5 [35].

In adults, MetS was defined [33,36,37,38,39] when 3 or more of the following factors were present: (1) abdominal obesity: defined in Asian population as a waist circumference ≥85 in men, and ≥80 cm in women and in a European population as a WC > 102 cm in men and >88 cm in women; (2) high blood pressure: systolic blood pressure ≥130 mmHg or diastolic blood pressure ≥85 mmHg or taking an antihypertensive drug treatment; (3) high fasting plasma glucose: ≥5.6 mmol/L (100 mg/dL) or on a drug treatment for type 2 diabetes; (4) hypertriglyceridemia: ≥1.7 mmol/L (150 mg/dL) or on a drug treatment for high plasma triglycerides; (5) reduced HDL-c: < 1.04 mmol/L (40 mg/dL) for men or <1.3 mmol/L (50 mg/dL) for women.

In children, MetS was considered when there was central obesity (WC ≥90th percentile or using the adult cut-offs if lower) along with the presence of ≥2 additional factors: (1) high serum triglycerides: ≥1.7 mmol/L; (2) high fasting plasma glucose: ≥5.6 mmol/L; (3) reduced HDL-c: ≤1.03 mmol/L; (4) high blood pressure: systolic blood pressure ≥130 mmHg or diastolic blood pressure ≥85 mmHg [40]. In the case of Kelishadi et al. [32], elevated blood pressure cut-offs were based on the 90th percentile of systolic and diastolic blood pressure according to gender, age, and height [41].

Diet quality was assessed by distinct methods, such as the following indexes or scores: healthy eating index [42,43], Mediterranean diet score [44,45], mean adequacy ratio [46,47], healthy diet indicator [45,48], dietary guidelines index [49] or dietary quality score [50].

## 3. Results

### 3.1. Study Inclusion

**Figure 1:**
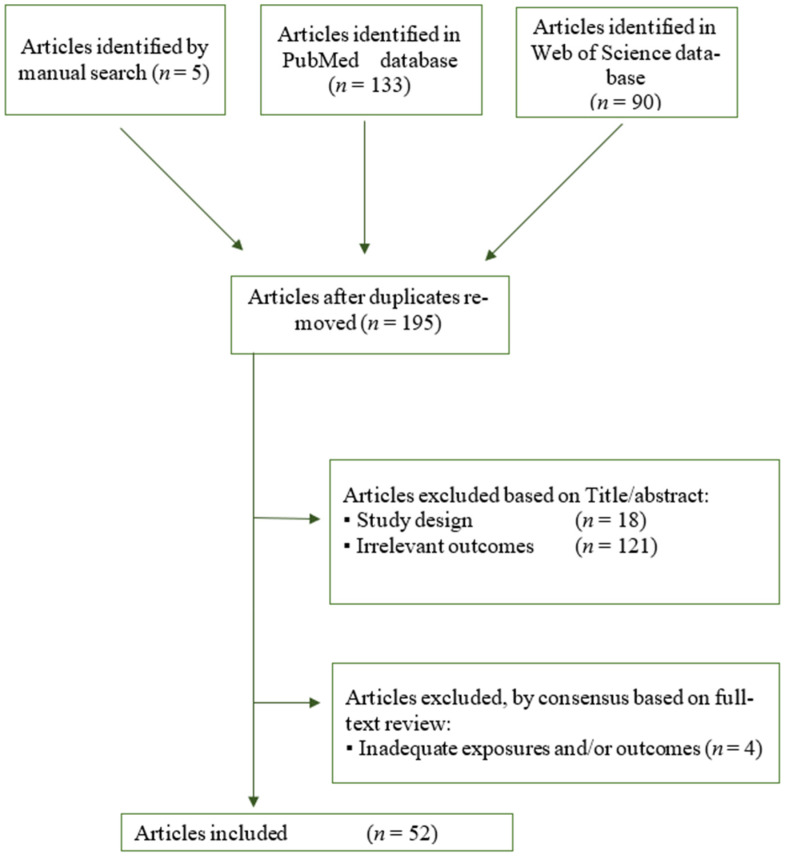
Flow diagram of the literature search and selection process.

The search identified 223 articles, 133 from PubMed and 90 from Web of Science. Additionally, five articles meeting inclusion criteria were identified from the manual search. Following an initial review of title and abstract, 176 articles were excluded: 33 duplicates, 121 did not present relevant outcomes, and 18 were reviews. Based on review of full manuscripts, an additional four articles were excluded by consensus due to irrelevant exposure or outcome. Therefore, 52 articles were included in this review (Figure 1).

### 3.2. Study Characteristic

Of the 52 studies, 18 were conducted in Asia, 14 in Europe, 18 in America, 1 in Oceania, and 1 in America and Europe. The year of publication spanned from 1964 to 2020. Nine studies (one study explored both eating speed/rate and eating frequency) included only children [4,12,14,24,26,27,28,30,31] one was based on children and adolescents (≤19 years-old) [51], two articles involved only adolescents [25,29], four articles recruited children, adolescents and young adults [10,15,17,32] and finally, 36 articles included only adults [2,5,9,11,13,16,18,19,20,21,22,23,52,53,54,55,56,57,58,59,60,61,62,63,64,65,66,67,68,69,70,71,72,73,74,75]. In total, 157,034 adults and 37,119 children and adolescents in the cross-sectional studies were included. Longitudinal studies incorporated 64,583 adults and 5610 children, and interventional trials were conducted in 357 adults and 24 children.

### 3.3. Cross-Sectional Studies

#### 3.3.1. Eating Speed/Rate

Table 1 shows the main characteristics and results from cross-sectional studies examining the associations between eating speed/rate with adiposity measures, metabolic syndrome, and diet quality.

##### Children

Regarding adipose measurements, fast eating speed/rate was significantly associated with greater risk of overweight (odds ratio (OR) = 2.71; 95% CI: 2.10, 3.48) and overweight/obesity (β = 0.70; 95% CI: 0.33, 1.08) [27,28], waist circumference [24] and higher BMI z-score [27]. Whereas, slowness in eating showed significantly inverse associations with overweight (OR = 0.61; 95%CI: 0.41, 0.92) and waist circumference [26]. No studies exploring dietary quality or metabolic risk biomarkers were found that met the inclusion criteria of this review.

##### Adults

From the total number of included articles exploring the association between eating speed and adiposity measures, four of them also examined MetS biomarkers, but none explored an association with dietary quality. Seven articles examined BMI, six looked at WC, and one assessed body weight. Hamada et al. [53] studied the speed of eating, subjectively (self-reported) and objectively (total number of chews, number of chews per bite, total meal duration, number of bites, chewing rate). They observed significant inverse correlations between total number of chews and total meal duration with body weight, BMI, waist circumference and abdominal obesity [53]. Moreover, several authors reported significant associations between self-reported fast eating speed/rate and increased risk for abdominal obesity prevalence [19,53,55], BMI [13,18,20,52,54], and larger WC [52,54]. No significant associations between eating speed and adiposity were reported in a study of a Spanish population conducted by Paz-Graniel et al. [21].

Regarding components of MetS, four studies explored associations between eating speed/rate and fasting plasma glucose, HDL-c, triglycerides, and blood pressure, and two studies assessed the risk of MetS prevalence. Paz-Graniel et al. [21] observed a 59% higher risk of hypertriglyceridemia in the fastest eating speed group compared to the slowest (HR = 1.59; 95% CI: 1.16–2.17). Similar results were reported in two additional studies [19,55], where fast-eating speed was associated with 7–69% greater risk of higher triglycerides. Fast eating speed/rate was also significantly associated with higher prevalence of low HDL-c [19,55] fasting plasma glucose [19], blood pressure [19], and MetS [19,55]. Nonetheless, not all cross-sectional studies in adults found significant associations between eating speed/rate and prevalence of high fasting plasma glucose [18,21,55] low HDL-c [18,21], high blood pressure [18,21,55], or MetS [21].

#### 3.3.2. Eating Frequency

Table 2 shows the description and relevant results from cross-sectional studies examining the associations between eating frequency with adiposity measures, MetS, and diet quality.

##### Children

The association between eating frequency and adiposity measures was explored in seven cross-sectional studies, four of these examined associations with BMI and waist circumference, three explored associations with BMI z-score, and two with total body weight. Significant inverse associations were reported between eating frequency and BMI [14,26,29,32], BMI z-score [14,25], body weight (β = −0.78) [14], WC [14,25,26], and abdominal obesity (OR = 0.73; 95% CI: 0.63, 0.85) [32]. In one study [14] an increase in eating frequency was significantly associated with higher BMI z-score in children with central obesity. In British and American adolescents, for every additional daily eating occasion or meal frequency, a significant increase in BMI z-score was observed [10,17]. No additional associations between eating frequency and anthropometric measures were reported in children [25,29,32].

MetS components as outcomes were examined in four cross-sectional studies. American and Finnish boys with higher eating frequency had significantly lower serum triglycerides concentrations (OR = 0.48; 95% CI: 0.26, 0.89) [29]. No additional associations with MetS components were found [10,25,29,32].

Evidence relating to diet quality in children and adolescents showed significant positive associations among eating frequency, meal frequency, or snack frequency and diet quality [15,51]. Other authors found significant negative associations with eating frequency [17], meal frequency [17], and snack frequency [17,51].

##### Adults

In total, 11 cross-sectional studies examined associations between eating frequency and adiposity in adults. A greater eating frequency was significantly associated with lower body weight [61], lower BMI [23,62,63,65] and lower waist circumference compared to a lower eating frequency [23,65]. Research conducted in British and Saudi participants [16,59,64] reported significant positive associations between snack frequency [16,59] or meal frequency [16] and the prevalence of overweight or obesity. In a study conducted by Fábry et al. [75], lower meal frequency was significant associated with higher overweight prevalence.

Individuals with high meal frequency [16] or snack frequency [16,64] have been reported to present with larger WC. In stratified analyses by weight status, men with BMI ≤ 25 kg/m^2^ were reported to have a negative association between snack frequency and WC (β = −0.52, 95% CI: −0.90, −0.14) [64]. This was not observed in women, however, in women with a BMI ≥ 25 kg/m^2^ a higher snack frequency was positively associated with WC (β = 0.80, 95% CI: 0.34, 1.26). In this study, women with waist circumference >88 cm showed a significantly greater snack frequency during the evening [64]. In South Korean participants, higher total eating frequency, but not the frequency of eating snacks, was significantly associated with lower BMI in participants with higher diet quality [23]. House et al. [63] reported that a significantly higher BMI z-score was observed in participants eating three or less meals per day compared to those eating four daily meals. Other authors [60,61,63] have reported no significant associations between eating frequency and BMI, body weight or WC. Kim et al. [22] reported that South Korean participants with a high frequency of eating and snacking and abdominal obesity had a 50% lower risk of having hypertension (OR = 0.5; 95% CI: 0.31, 0.82). Participants with high snack frequency and low diet quality also showed 50% lower risk of hypertension (OR = 0.5; 95% CI: 0.23, 0.89) compared to those with no snack consumption.

Seven cross-sectional studies assessed associations between meal or snaking frequency and diet quality. Significant positive associations were observed between eating frequency, meal frequency or snack frequency and diet quality in six studies [11,16,57,58,65,66]. A significant but negative association between diet quality and distinct assessment methods for snack frequency have also been reported [16]. In one study, no associations between these exposures and diet quality were found [60].

### 3.4. Longitudinal Studies

#### 3.4.1. Eating Speed/Rate

Table 3 shows longitudinal studies exploring the associations between eating speed/rate and adiposity outcomes.

##### Children

Only one longitudinal study met inclusion criteria. After 1 year of follow-up, Okubo et al. [28] reported a positive association between rate of eating at 30 months of age and overweight or obesity at 42 months of age (β = 0.67; 95% CI: 0.24–1.10).

##### Adults

All of the longitudinal studies included explored adiposity measures but only one examined MetS components. Tanihara et al. [9] and Yamane et al. [2] observed that a fast eating speed/rate was associated with a significantly increased risk of being overweight, however in another study conducted in New Zealand [56] no significant associations were reported. Fast eating speed/rate was also associated with a 35% increased risk of developing increased WC (hazard ratio (HR) = 1.35; 95% CI: 1.10–1.66) [5], a 37% higher risk for developing low HDL-c (HR = 1.37; 95% CI: 1.12–1.67) and were 30% more likely to experience MetS incidence (HR = 1.30; 95% CI: 1.05–1.60) in a Japanese population [5].

#### 3.4.2. Eating Frequency

Table 4 shows the characteristics of the longitudinal studies included examining the associations between eating frequency and adiposity, MetS or diet quality.

##### Children

Three longitudinal studies explored the association between eating frequency and anthropometric measures. In two studies, each with a 10-year follow-up, eating frequency was shown to be significantly inversely associated with WC [30], BMI z-score [31] and BMI [30]. Ritchie et al. [30], reported that greater meal frequency was associated with higher BMI in American female adolescents. Additional associations for BMI z-score and overweight were not observed [4,31].

##### Adults

Two longitudinal articles exploring the association of eating frequency with adiposity met inclusion criteria. Kahleova et al. [67] conducted a 7-year follow-up study, reporting a positive association between eating frequency and changes in BMI (β = 0.04; 95% CI: 0.02–0.06). Whereas, Larsen et al. [74] observed that higher baseline meal frequency was associated with a decrease in BMI (β = −0.14; 95% CI: −0.27–0.00) and waist circumference (β = −0.49; 95% CI: −0.99–0.00) during a 6-year follow-up [74].

### 3.5. Interventional Trials

#### 3.5.1. Eating Speed/Rate

Table 5 summarizes the details of the intervention trials that met inclusion criteria assessing the effect of eating speed/rate on adiposity measures, metabolic syndrome, and diet quality.

##### Children

Only one intervention trial met inclusion criteria. Faith et al. [12] conducted an 8-week parallel study in 24 American children aged 4 to 8 years old. The participants in the intervention group attended interactive sessions focused on decelerate eating speed and were encouraged to eat slower through the use of timers and the performance of interactive activities with their families during eating occasions. The eating speed decreased in the intervention group and was associated with significantly lower BMI and BMI z-score.

##### Adults

No intervention trials met inclusion criteria assessing eating speed/rate in adults.

#### 3.5.2. Eating Frequency

Table 6 shows the characteristics and relevant results from intervention articles investigating the effect of eating frequency on adiposity measures, MetS, and diet quality.

##### Children

No intervention trials article met inclusion criteria assessing eating frequency in children.

##### Adults

Three intervention trials investigated the effect of eating frequency on measures of adiposity. Stote et al. [72] conducted a crossover study in Americans, where the intervention consisted of either one or three daily meals for two 8-week periods. Compared with one daily meal, the group consuming the three daily meals pattern had a significantly higher body weight. In a Turkish study, different dietary patterns showed no effects for WC or BMI [73]. Similarly, an American trial studying meal frequency also did not observe a significant effect on body weight, WC or BMI [71]. Diet quality was not examined in any intervention trial that met inclusion criteria for adults.

The impact of eating frequency on MetS components was examined in seven intervention trials. Stote et al. [72] reported that greater meal frequency resulted in a significantly lower blood pressure and HDL-c. An American study [69], comparing consumption of two or six daily meals for a total of 6 weeks reported that low-eating frequency induced higher HDL-c levels. In an American population, compared with one daily meal, three meals per day led to a significantly lower fasting plasma glucose in a study that lasted 18 weeks [70]. Kanaley et al. [68] conducted a study in Americans in which the main interventions were a dietary pattern consisting of three meals, six meals or six high protein meals per day. Compared to the dietary patterns involving three meals or six meals, eating six high protein daily meals led to lower levels of fasting plasma glucose [68]. Other studies [72,73] reported no significant effects of different eating frequency patterns on MetS components.

## 4. Discussion

The findings of this review suggest that a faster eating speed/rate could be associated with an increased risk of adiposity and MetS or its components. Furthermore, a greater eating frequency may be mainly associated with diet quality and lower risk of adiposity and MetS or its components.

Regarding eating speed/rate in children, some cross-sectional studies concluded that eating speed was positively associated with overweight [26,27] and positively correlated to WC [24]. Similar results were also reported in a longitudinal [28] and in an interventional trial [12]. Associations between eating frequency and adiposity (BMI, BMI z-score, waist circumference and body weight) in children were mainly inversely associated in cross-sectional [25,26,29] and longitudinal studies [30,31]. However, some authors reported positive associations between eating frequency and BMI z-score in adolescents [10,17] and in children with central obesity [14]. In adults self-reported faster eating speed was frequently associated with higher risk of overweight or obesity and larger WC [13,20] in cross-sectional studies but only overweight was associated with faster eating speed/rate in longitudinal studies [2,9]. However, an observational study reported positive partial correlations between objective eating speed with BMI, body weight, and WC [53]. Regarding eating frequency, inverse associations were reported in cross-sectional and longitudinal studies with adiposity indicators [74,75], except for a few observational and interventional studies that otherwise reported positive associations [16,64,72]. Differences in the findings among studies could be partly explained by gender-specific lifestyle factors such as body composition, physical activity, hormone function, and dietary habits [76]. Moreover, cardiovascular risk factors may coexist simultaneously, increasing the risk of developing additional related metabolic alterations as a result of synergic effects [19]. In childhood, it has been proposed by some authors that higher parenthood control [77,78] and consequently feeding restriction, as well as an improvement in energy compensation, which declines with age [14] may contribute to disparities in adiposity, BMI z-score, or body weight for age until 5 years old [79].

With regard to diet quality, few cross-sectional studies reported that higher eating frequency, snack frequency and meal frequency improved diet quality in American children and adolescents [15,51]. On the contrary, inverse associations between snack frequency and diet quality were reported mainly in teenagers [17,51]. Similar to in adulthood, more autonomy in food choices prevail in adolescence, and this may play an important role in the quality of the diet, especially in regard to snacking. [51]. In adults, higher diet quality was mainly positively associated with the number of eating occasions [16,57,58,65]. However, one author reported inverse results specifically for snack frequency [16]. It should be noted that snack frequency was usually defined as the number of eating occasions outside of main meals [16] and that differed from meals based on the amount of energy contributed. Nowadays a harmonized, globally accepted criterion for diet quality has not been defined, perhaps due to differences in culture and traditions. However, several organizations have published dietary guidelines that describe specific indicators, groups of food or even healthy dietary patterns, which may be used to fit *a priori* diet quality indexes as were described in the articles included in this review.

With reference to MetS and its components, observational studies conducted in adults showed that eating speed/rate was significantly associated with MetS onset [5,19,55], high fasting plasma glucose, triglycerides, blood pressure [18,19,21], and low HDL-c [18,55]. In contrast, distinct assessment methods of eating frequency were inversely associated with lower risk of hypertension in adults with abdominal obesity or with lower diet quality [22]. Furthermore, in interventional trials, a greater eating frequency was associated with lower fasting plasma glucose [68,70].

The potential mechanism by which eating behaviors (eating speed/rate or eating frequency) are linked to adiposity and cardiometabolic risk factors remain unclear. Eating is a complex physiological act influenced by multiple endogenous and exogenous factors. Previous studies highlight mastication [27], bite frequency [80,81], oral sensory exposure to meals and food texture as potential determinants for feeding regulation, chiefly by inducing gastrointestinal hormonal secretion [82,83]. In this process, numerous gastrointestinal hormones are involved but mainly ghrelin, peptide YY, leptin and insulin [82], are noted to play a key role in energy intake, [84] adiposity and metabolism [85]. Leptin and insulin are classified as long-acting adiposity signals due to their role in lipid and glucose metabolism [86], with an important influence on adiposity regulation [82]. While ghrelin determines meal initiation and peptide YY induces a decrease in appetite [87]. In fact, an increase in peptide YY has been reported with slowing eating speed [83]. Additionally, it has been suggested that longer oro-sensory exposure may promote greater satiation and therefore, a protective effect on overfeeding [24]. Moreover, eating frequency has been related to satiety [88] and thermic effect of food, which has the potential to impact metabolic rate [63]. Additionally, a lower eating frequency has been associated with reduced insulin sensitivity caused by a higher insulin response to meals in irregular eaters [22]. This is related to overactivation of the sympathetic nervous system, excess of angiotensinogen secretion and renal sodium retention with evident effects in blood pressure.

Despite the potential effects of eating behaviors (eating speed and eating frequency), it is already known that the onset of overweight, obesity, and MetS is the result of a multifactorial etiology [89], such as gene–environment factors [3] (including weight gained during pregnancy [29] or parental BMI [28]), type of population [32], sleep duration [29], ethnicity [90], parental income [26] and physical activity [30]. Furthermore, we cannot disregard the influence of differences in methodology (follow-up periods, intervention group, age, socioeconomic status, total energy intake, cultural differences in dietary habits, etc.) among studies that might explained the discrepancies observed in the present findings.

In the present review we sought to synthetize the available evidence regarding eating behaviors in relation to adiposity, MetS components and diet quality. However, this review is not without limitations. First, due to its design, cause–effect associations in cross-sectional and longitudinal studies are not possible to discern and we cannot disregard that relevant articles may exist that were not included. Secondly, drawing firm conclusions is difficult due to the great variability and heterogeneity between articles, the lack of consensus about eating speed/rate and eating frequency definitions, as well as differences in overweight or obesity definitions used for children. Third, eating speed/rate was explored, in the vast majority of studies, subjectively through self-reported questionnaires, which may not reflect true eating speed/rate [53]. Moreover, BMI was used frequently to assess adiposity despite its several limitations [91]. Similarly, it is important to highlight that one study used an out-of-date definition of overweight [75]. A special consideration about the criteria used to define metabolic syndrome is that is has undergone several variations since 1999. However, all of the articles included in the present review used the harmonized criteria established by Alberti et al., in 2009 except for one study which used the ATPIII criteria. Likewise, it is necessary to highlight the limited number of studies conducted in children, and the scarcity of longitudinal and interventional trials exploring associations between eating speed/rate or meal frequency and diet quality.

## 5. Conclusions

Limited evidence suggests an association between faster eating speed/rate, adiposity and increased risk of developing MetS. While a higher eating frequency is associated with lower adiposity, better diet quality and lower MetS risk. However, more long-term and interventional trials are warranted in the future to clarify these associations and the mechanisms by which they affect adiposity and cardiometabolic health. If these associations were proven, strategies focused on eating behaviors in early life, such as eating speed/rate and eating frequency may be recommended for the prevention of excess body weight, cardiovascular risk, and metabolic disease development.

## Figures and Tables

**Table 1:** Characteristics and main findings of cross-sectional studies that explored eating speed/rate.

Cross-Sectional Studies					
**Children**					
**Author and Year**	**Population**	**Exposure (s)**	**Outcome (s)**	**Adjusted Variables**	**Results**
Eloranta 2012 [26].	▪ PANIC study. ▪ Finnish. ▪ *n* = 510 (247 girls and 263 boys). ▪ Age: 6–8 years.	Slowness in eating: finishing eating in more than 30 min (self-reported).	OW, OB, WC, and HC.	Sex, age, total daily time of PA, total daily screen time, and parental income level.	▪ Slowness in eating: OW/OB (OR = 0.61 [95% CI: 0.41, 0.92]), WC (β = −0.16, *p* < 0.01) and HC (β = −0.17, *p* < 0.01).
Fogel 2017 [24].	▪ GUSTO cohort. ▪ Asian. ▪ *n* = 386 (184 girls and 202 boys). ▪ Age: 4.5 years.	Eating rate (video recorded—gr/min).	WC.	None.	▪ Eating rate: WC (r = 0.17, *p* < 0.01).
Okubo2017 [28].	▪ Osaka Maternal and Child Health Study. ▪ Japanese. ▪ *n* = 492 mother-child pairs. ▪ Age: 30–42 m. ▪ 1 year follow-up.	Eating rate (self-reported).	BMI.	Child’s sex and age (at fourth survey), maternal age and BMI at enrollment, education level, family income, pregnancy smoking status, maternal working status at 30 months postpartum, birth order, birth weight, duration of breast-feeding, time spent watching TV at 30 months of age, protein intake, fat intake, and dietary fiber at 30 months of age.	Fast eating rate vs. slow eating rate at 30 m.: ▪ BMI at 30 m. (β = 0.70 [95% CI: 0.33, 1.08]).
Okubo 2018 [27].	▪ SHOKUIKU Study ▪ Japanese ▪ *n* = 4451 (2136 girls and 2315 boys). ▪ Age: 5–6 years.	Eating rate (self-reported).	OW and BMI z-score.	Sex, age, n° of siblings, PA and birthweight; residential block, parents’ educational attainment, parents weight status, protein (% of energy), fat (% of energy), and dietary fiber intake (g/1000 kcal).	▪ Fast eating rate vs. medium eating: OW (OR = 2.71 [95% CI: 2.10, 3.48]). ▪ Fast eating rate: >BMI z-score (*p* < 0.01).
**Adults**					
**Author and Year**	**Population**	**Exposure (s)**	**Outcome (s)**	**Adjusted Variables**	**Results**
Lee 2013 [18].	▪ South Korean. ▪ *n* = 8775 (3956 women, 4819 men). ▪ Age: 20–80 years.	Eating rate (self-reported).	High FPG, high BP, low HDL-c, high TG, BMI (>25 kg/m^2^).	Age, alcohol, smoking, and exercise and BMI, total energy intake.	Fast eating rate vs. slow eating speed: ▪ Women: BMI (OR = 3.35 [95% CI: 2.23, 5.3]). Other variables: NA.
Nagahama 2014 [55].	▪ Japanese. ▪ *n* = 56,865 (15, 045 women and 41,820 men). ▪ Age: 17–99 years.	Self-reporting eating rate.	MetS, CO, high BP, high FPG, high TG, low HDL-c.	Age, smoking status, alcohol, regular physical activity and body mass index.	Fast eating rate vs. normal eating rate: ▪ Men: CO (OR = 1.97 [95% CI: 1.88, 2.07]), low HDL-c (OR = 1.10 [95% CI: 1.03, 1.18]), high TG (OR = 1.07 [95% CI: 1.02, 1.12]) and MetS (OR = 1.10 [95% CI: 1.03, 1.17]). ▪ Women: CO (OR = 1.44 [95% CI: 1.33, 1.56]). Other variables: NA.
Lee 2016 [20].	▪ Japanese. ▪ *n* = 4249 (2163 women and 2086 men). ▪ Age: 20–80 years.	Eating quickly (self-reported).	OW.	Gender, age, living with spouse, occupation, education, visiting hospitals, habitual exercise, smoking status, and alcohol drinking.	▪ Eating quickly vs. no eating quickly: OW (OR = 1.92 [95% CI: 1.62, 2.28]).
Hamada 2017 [53].	▪ Japanese. ▪ *n* = 84 (women college students). ▪ Age: 19 years.	Eating speed: - Subjective (self-reported) - Objective (total number of chews, number of chews/bites, total meal duration, number of bites, chewing rate).	BW, BMI, WC, AC and HC.	None.	▪ Total # chews and meal duration (p < 0.05): BW (r = 0.22, 0.24), BMI (r = 0.24, 0.27), WC (r = 0.26, 0.24), AC (r = 0.25, 0.27) and HC (r = 0.24, 0.22). ▪ Total # bites (p < 0.05): BW (r = 0.25) and AC (r = 0.25). ▪ Subjective fast eating speed vs. subjective slow eating speed (p < 0.05): > BW, BMI, WC, AC and HC.
van den Boer 2017 [13].	▪ Dutch. ▪ *n* = 1473 (732 women and 741 men). ▪ Age: 20–70 years.	Eating speed (self-reported).	OW.	Age, smoking, level of education, emotional eating, restrained eating, external eating, energy intake, moderate to vigorous activity, and sedentary activity.	Fast eating speed vs. average eating speed in total population: OW (β = 0.90 [95% CI: 0.48, 1.32]). ▪ Stratified analysis: OW in women (β = 1.13 [95% CI: 0.43, 1.84]).
Sonoda 2018 [52].	▪ Japanese. ▪ *n* = 863 men (From Japanese Maritime Self Defense Force).	Eating speed (self-reported).	BMI and WC.	Eating speed, n° of missing functional teeth, periodontal status, age, military ranks, alcohol, smoking, and exercise frequency.	▪ Fast eating speed vs. slow/very slow eating speed: BMI≥ 25 kg/m^2^ (OR = 5.04 [95% CI: 1.95, 13.07]), BMI≥ 30 kg/m^2^ (OR = 4.80 [95% CI: 1.21, 19.09]), WC≥ 85 cm (OR = 6.59 [95% CI: 2.37, 18.48]) and WC≥ 90 cm (OR = 5.22 [95% CI: 1.81, 15.06]).
Tao 2018 [19].	▪ Chinese. ▪ *n* = 7972 (3508 women and 3436 men). ▪ 18–65 years.	Eating speed (self-reported).	MetS, CO, elevated BP, elevated FPG, elevated TG, reduced HDL-c.	Age, education level, work stress, PA intensity, PA frequency, sleep duration, smoking, drinking, high salt intake, high intake of sugar, fat and/or meat, a mainly vegetable diet, frequency of eating breakfast, grain consumption, antihypertensive, antidiabetic, and/or hypolipidemic medication.	Fast eating speed vs. slow eating speed: ▪ Total population: prevalence for MetS (OR = 2.27 [95% CI: 1.80, 2.86]), CO (OR = 1.68 [95% CI: 1.35, 2.09]), BP (OR = 1.82 [95% CI: 1.48, 2.24]), TG (OR = 1.51 [95% CI: 1.21, 1.88]) and HDL-c (OR = 1.33 [95% CI: 1.11, 1.60]). ▪ Men: prevalence for MetS (OR = 2.21 [95% CI: 1.69, 2.91]), CO (OR = 1.53 [95% CI: 1.16, 2.02]), BP (OR = 1.50 [95% CI: 1.17, 1.92]), TG (OR = 1.69 [95% CI: 1.29, 2.23]) and HDL-c (OR = 1.35 [95% CI: 1.02, 1.79]). ▪ Women: prevalence for MetS (OR = 2.27 [95% CI: 1.46, 3.53]), CO (OR = 1.98 [95% CI: 1.36, 2.88]), BP (OR = 3.02 [95% CI: 2.00, 4.56]) and FPG (OR = 1.56 [95% CI: 1.05, 2.33]).
Paz-Graniel 2019 [21].	▪ PREDIMED-Reus study. ▪ Spanish. ▪ *n* = 792 (451 women and 341 men). ▪ Age: 55–80 years.	Eating speed (self-reported).	OB, MetS, CO, hypertriglyceridemia, low HDL-c, high BP, high FPG.	Age, sex, educational level, smoking status, use of dental prosthesis, total energy intake (kcal/day), alcohol consumption (g/day), physical activity (MET/min/day), and adherence to Mediterranean diet.	▪ Fast eating speed: HR+ 59% for hypertriglyceridemia (HR = 1.59 [95% CI: 1.08, 2.02]). ▪ MetS and other variables: NA.
Wuren 2019 [54].	▪ Japan Multi-Institutional Collaborative Cohort study. ▪ Japanese. ▪ *n* = 5888 (2495 women and 3393 men). ▪ Age: 70–79 years.	Eating rate (self-reported).	BMI (> 25 kg/m^2^) and WC (> 80 cm in females and > 90 cm in males).	Age, current smoker, alcohol, PA, total energy intake, medication for hypertension, diabetes and/or dyslipidemia, sleep duration, psychological stress, education level, family structure, fast food, restaurants or food service use, packed lunch, dinner, snacking, and breakfast time.	Fast eating rate vs. normal eating rate: ▪ BMI (men: (OR = 1.48 [95% CI: 1.25, 1.76]; women: OR = 1.78 [95% CI: 1.39, 2.26]). ▪ WC (men: OR = 1.45 [95% CI: 1.21, 1.74]; women: OR = 1.34 [95% CI: 1.11, 1.61]).

Abbreviations: AC, abdominal circumference; β, beta coefficient; BMI, body mass index; BP, blood pressure; BW, body weight; CO, central obesity; FPG, fasting plasma glucose; GUSTO, Growing UP in Singapore Towards Healthy Outcomes; HC, hip circumference; HDL-c, high density lipoprotein cholesterol; HR, hazard ratio; M., months; MET, Metabolic Equivalent of Task; MetS, metabolic syndrome; NA, no associations; OB, obesity; OR, odds ratio; OW, overweight; PA, physical activity; r, PANIC, Physical Activity and Nutrition in Children; Pearson’s correlation; PREDIMED, Prevencion con Dieta Mediterránea; TG, triglycerides; vs., versus; WC, waist circumference.

**Table 2:** Characteristics and main findings of cross-sectional studies that explored eating frequency.

Cross-Sectional Studies					
**Children**					
**Author and Year**	**Population**	**Exposure (s)**	**Outcome (s)**	**Adjusted Variables**	**Results**
Eloranta 2012 [26].	▪ PANIC study. ▪ Finnish. ▪ *n* = 510 (247 girls and 263 boys). ▪ Age: 6–8 years.	Eating frequency: ▪ 3 main meals/day: breakfast, lunch and dinner. ▪ Snacks (all eating and drink occasions besides main meals).	OW/OB, WC, HC.	Sex, age, total daily time of physical activity, total daily screen time, and parental income level.	Eating 3 main meals vs. not eating 3 main meals: ▪ OW/OB (OR = 0.37 [95% CI: 0.18, 0.75]). ▪ WC (β = −0.16, *p* < 0.01) and HC (β = −0.1, *p* < 0.01). Other variables: NA.
Jennings 2012 [14].	▪ SPEEDY study. ▪ British. ▪ *n* = 1700 (952 girls and 748 boys). ▪ Age: 9–10 years.	Eating frequency: number of time periods of food or drinks consumption (6–9 h, 9–12 h, 12–14 h, 14–17 h, 17–20 h, 20–22 h, 22 h–6 h): ▪ Breakfast (6–9 h). ▪ Mid-day meal (12–14 h). ▪ Evening meal (17–20 h). ▪ Snack (any other time).	BMI, BMI z-score, BW and WC.	Gender, parental education, under-reporting, energy intake, and physical activity.	▪ Healthy weight children (*p* ≤ 0.03): BW (β = −0.78), BMI (β = −0.17), BMI z-score (β = −0.10) and WC (β = −0.38). ▪ Centrally obesity children: BMI z-score (β = 0.09, *p* < 0.05). ▪ Other variables: NA.
Jääskeläinen 2013 [29].	▪ Northern Finland Birth Cohort 1986. ▪ Finnish. ▪ *n* = 6247 (3181 girls and 3066 boys). ▪ Age: 16 years.	Meal frequency: ▪ Regular meal pattern: 5 meals a day including breakfast. ▪ Semi-regular meal pattern: ≤4 meals a day including breakfast. ▪ Breakfast skippers: ≤ 4 meals a day, not including breakfast.	OW/OB, WC, hyperglycemia, hypertriglyceridemia, low HDL-c, HT.	Early life factors: birth weight for gestational age, maternal weight gain in the first 20 weeks of gestation, maternal pre-pregnancy BMI, pregnancy smoking, maternal glucose metabolism, and parity. Later childhood factors: tobacco, sleep duration, PA, sedentary time, Tanner stage, parental education level, and body mass index.	Regular meal pattern vs. semi-regular meal pattern: 1. Model adjusted for early life factors: ▪ Boys: hypertriglyceridemia (OR = 0.48 [95% CI: 0.26, 0.89]) and OW/OB (OR = 0.47 [95% CI: 0.34, 0.65]). ▪ Girls: OW/OB (OR = 0.57 [95% CI: 0.41, 0.79]). 2. Model adjusted for later childhood factors: ▪ Boys: OW/OB (OR = 0.41 [95% CI: 0.29, 0.58]). ▪ Girls: OW/OB (OR = 0.63 [95% CI: 0.45, 0.89]). Other variables: NA.
Murakami 2014 [10].	▪ British. ▪ *n* = 1636 (803 girls and 833 boys). ▪ Age: 4–18 years.	Eating frequency: all eating occasions for food/drinks, except for those providing < 210 kJ of energy.	BMI z-score, HDL- c, TG, SBP and DBP.	Age, sex, social class, physical activity levels, intakes of protein, fat, total sugar and dietary fiber and BMI z-score (in the analysis of blood lipid profile, and blood pressure).	▪ Adolescents ≥ 11 years: BMI z-score (β = 0.11, *p* < 0.01). ▪ Other variables: NA.
Evans 2015 [51].	▪ Daily D study. ▪ American. ▪ *n* = 176 (89 girls and 87 boys). ▪ Age: 9–15 years.	Eating, meal and snack frequency.	DQ (HEI-2005).	School, maternal education, free or reduced-price school lunch eligibility, and physical activity.	In children (9–11 years.): ▪ EF (β = 2.60, *p* < 0.01) and SF (β = 2.31, *p* = 0.02). In adolescents (12–15 years.): ▪ MF (β = 5.40, *p* = 0.01) and SF (β = −2.73, *p* < 0.01).
House 2015 [25].	▪ SOLAR cohort. ▪ American (Hispanic). ▪ *n* = 191 (83 girls and 108 boys). ▪ Age: 11–15 years.	Eating frequency: ▪ Infrequent: <3 per day. ▪ Frequent: ≥3 meals/day.	BMI z-score, WC, BW, FPG, HDL-c, TG.	Tanner stage, sex, mean energy, total fat, total fat and height, total lean, and height and insulin sensibility.	▪ Frequent eaters vs. Infrequent eaters (*p* ≤ 0.01): <BMI z-score, WC and TG. ▪ Other variables: NA.
Kelishadi 2016 [32].	▪ CASPIAN-IV study. ▪ Iranian. ▪ *n* = 13,486 (6635 girls and 6851 boys). ▪ Age: 6–18 years.	Eating frequency (≤3, 4, 5 or ≥6): ▪ Breakfast/lunch/dinner. ▪ Number of snacks.	Elevated BP, elevated DBP, elevated SBP, OW, OB, and AO.	Age, gender, and living area, Screen time, physical activity, socioeconomic status, and sleeping hours, and BMI (only in blood pressure).	▪ ≥6 EF vs. ≤ 3 EF: OB (OR = 0.54 [95% CI: 0.44, 0.65]) and AO (OR = 0.73 [95% CI: 0.63, 0.85]). ▪ Other variables: NA.
Murakami 2016 [17].	▪ British. ▪ *n* = 1636 (803 girls and 833 boys). ▪ Age: 4–18 years.	1. Eating frequency: times/day. 2. Meal and snack frequencies: ▪ Based on energy percentage contribution (meal with ≥15% and snack with <15% of total energy). ▪ Based on time (meals: 6–10 h, 12–15 h and 18–21 h; snack: any other time).	DQ (MDS) and BMI z-score.	Age, sex, social class, physical activity and plausible energy reporters (in adolescents).	In children (4–10 years.): ▪ DQ (*p* < 0.01): EF (β = −0.30), SF-energy% (β = −0.20) SF-time (β = −0.31), MF-time (β = −0.40). In adolescents: ▪ DQ (*p* ≤ 0.03): EF (β = −0.10), SF-energy-% (β = −0.09), SF-time (β = −0.18) ▪ BMI z-score: MF-clock time (β = 0.13, *p* = 0.02).
Murakami 2016 [15].	▪ American. ▪ *n* = 10,462 (5188 girls and 5274 boys). ▪ Age: 6–19 years	Eating frequency: times/day. Meal and snack frequencies: ▪ Self report (meals: breakfast, brunch, lunch, supper, and dinner; others: snacks) ▪ Based on time (meals: 6–9 h, 12–14 h and 17–20 h; snack: other). ▪ Based on energy percentage contribution (meal: ≥15%, snack: <15% of total energy).	DQ (HEI-2010).	Sex, age, race/ethnicity, family poverty income ratio, education of household head, household size, PA, watching television and computer use, weight status, dietary reporting status, and survey cycle.	Children of 6–11 years. (*p* < 0.03): ▪ EF (β = 1.21), MF-energy% (β = 1.45), MF-self report (β = 3.59) and MF-time (β = 1.72), SF-energy% (β = 0.70) and SF-self report (β = 0.60). Adolescents (*p* < 0.01): ▪ EF (β = 1.52), MF-energy% (β = 1.74), MF-self report (β = 3.56) and MF-time (β = 1.99) and SF-energy% (β = 1.00).
**Adults**					
**Author and Year**	**Population**	**Exposure (s)**	**Outcome (s)**	**Adjusted Variables**	**Results**
Fábry 1964 [75].	▪ Czech. ▪ *n* = 379 men. ▪ Age: 60–64 years.	Meal frequency: ≤ 3, 3–4 (±snacks) or ≥ 5 per day	OW.	None.	▪ ≤3 MF vs. ≥5 MF: > OW (*p* < 0.05).
Drummond 1998 [61].	▪ Scottish. ▪ *n* = 79 (37 women and 42 men). ▪ Age: 20–55 years.	Eating frequency: ▪ Eating occasion (food and beverage). ▪ Meal (breakfast, lunch or dinner). ▪ Snack.	BW and BMI.	None.	▪ EF in men: BW (r = −0.34, *p* = 0.03). ▪ Other variables: NA.
Zizza 2012 [66].	▪ American. ▪ *n* = 11,209 (5789 women and 5420 men. ▪ Age: ≥ 20 years.	Snack frequency: 0, 1, 2, 3 o ≥ 4.	DQ (HEI-2005).	Sex, race/ethnicity, education, smoking, PA, consumption of ≥ meals/day, chronic diseases, age, BMI, and meals energy contribution.	▪ Higher SF: > DQ (*p* < 0.01).
Kim 2014 [22].	▪ South Korean. ▪ *n* = 4625 (2294 women and 2331 men). ▪ Age: ≥ 19 years.	▪ Eating frequency: all eating occasions (<2, 3, 4 or ≥5). ▪ Meal frequency: 1, 2 or 3. ▪ Snack frequency: 0, 1, 2 or ≥ 3.	HT, AO (WC ≥85 cm in women and ≥90 cm in men) and DQ (MAR).	Age, sex, smoking, smoking amount, alcohol consumption frequency, PA frequency, IPAQS, total daily calorie/sodium/potassium/calcium intake, sleep sufficiency, stress level, MAR, BMI, WC, meal, and snack frequency.	≥5 EF vs. 3 EF: ▪ HT in AO (OR = 0.5 [95% CI: 0.31, 0.82]). ≥3 SF vs. 0 EF: ▪ HT in DQ < 50% (OR = 0.5 [95% CI: 0.23, 0.89]).
Aljuraiban 2015 [62].	▪ INTERMAP study. ▪ American and British. ▪ *n* = 2385 (1153 women and 1232 men). ▪ Age: 40–59 years.	Eating frequency/day: < 4, 4 to < 5, 5 to < 6 and 6.	BMI.	Age, gender, educational level, hours of moderate and heavy physical activity, smoking, special diet, dietary supplement use, and population sample.	▪ BMI (β = −1.1 [95% CI: −1.6, −0.7]).
O’Connor 2015 [64].	▪ Fenland study. ▪ British. ▪ *n* = 10,092 (5446 women and 4646 men).	Snack frequency (self-reported).	BMI and WC.	Age, alcohol, smoking, age at completing full-time education, test site, main meal, light meal, drink-only snack, plasma vitamin C, energy intake, screen time, and PA energy expenditure.	SF in women: BMI (β = 0.29 [95% CI: 0.13, 0.44]) and WC (β = 0.73 [95% CI: 0.4, 1.1]). Every additional unit in SF: ▪ Women with BMI ≥ 25 kg/m^2^: WC (β = 0.80 [95% CI: 0.34, 1.26]). ▪ Men with BMI < 25 kg/m^2^: WC (β = −0.52 [95% CI: −0.90, −0.14]).
Barnes 2015 [60].	▪ American. ▪ *n* = 233 (157 women and 76 men). ▪ Age: 18–60 years.	Snack frequency: Beverage and/or meal in any eating occasion that is not breakfast, lunch, or dinner.	BMI and DQ (HEI-2010).	Age, sex, race/ethnicity, education level, income, job type, marital/partner status, physical activity, and total daily energy intake.	▪ No significant associations.
Holm 2015 [58].	▪ European. ▪ *n* = 7531 (3612 women and 3919 men). ▪ Age: 15–80 years.	Meal frequency: 0–3, 4, ≥5.	DQ (DQS).	Age, gender, household composition, educational level, occupational status and socioeconomic level.	▪ EF ≥ 5 vs. EF 0–3 (*p* ≤ 0.02): Denmark (β = 0.71), Finland (β = 0.37), Norway (β = 0.48) and Sweden (β = 0.68).
Zhu 2016 [65].	▪ American. ▪ *n* = 7791 (3774 women and 4017 men). ▪ Age: ≥ 20 years.	Eating frequency (self-reported).	DQ (HEI-2010), BMI, WC.	Age, race/ethnicity, ratio of family income: poverty, PA level, smoking, and energy intake.	Both genders: ▪ DQ in women (β = 1.63, *p* < 0.01) and men (β = 1.7, *p* < 0.01). ▪ WC in women (β = −0.8, *p* = 0.01) and men (β = −0.6, *p* = 0.03). Women: ▪ BMI (β = −0.4, *p* < 0.01).
Murakami 2016 [16].	▪ British. ▪ *n* = 1487 (809 women and 678 men). ▪ Age: 19–64 years.	Meal and snack frequency: ▪Based on energy percentage contribution: meal: ≥15% of total energy intake; snack: < 15%. ▪ Based on time: meal: from 06.00–10.00 h, 12.00–15.00 h or 18.00–21.00 h; snack: other occasion.	DQ (MDS and HDI), BMI and WC.	Age, social class, EI: EER (energy intake misreporting), smoking status, PA, protein intake (% of energy), fat intake (% of energy), total sugar intake (% of energy), alcohol intake (% of energy), and dietary fiber intake (g/10 MJ, continuous).	MDS or HDI: Men ▪ Positive (*p* ≤ 0.02): MF-energy% (MDSβ = 0.27, HDIβ = 0.26). ▪ Negative (*p* < 0.01): SF-energy% (MDSβ = −0.12, HDIβ = −0.09), SF-time (MDSβ = −0.19, HDIβ = −0.17). Women ▪ Positive (*p* < 0.05): MF-time (MDSβ = 0.14, HDIβ = 0.12), MF-energy% (MDSβ = 0.27). ▪ Negative (*p* ≤ 0.02): SF-time (MDSβ = −0.36, HDIβ = −0.15), SF-energy% (MDSβ = −0.11). BMI or WC: Men ▪ Positive (*p* < 0.05): MF-time (BMIβ = 0.33, WCβ = 0.85), SF-time (BMIβ = 0.36, WCβ = 0.87) and SF-energy% (BMIβ = 0.32, WCβ = 0.77).Women ▪ Positive (*p* ≤ 0.02): SF-time (BMIβ = 0.88, WCβ = 2.09) and SF-energy% (BMIβ = 0.4, WCβ = 0.69).
Murakami 2016 [11].	▪ American. ▪ *n* = 19,427 (9826 women and 9601 men). ▪ ≥20 years.	Eating frequency: all eating occasions (kcal >50). Meal and snack frequencies: ▪ Self-reported (meals: breakfast, brunch, lunch, and dinner; others: snacks) ▪ Based on time (meals: 6–9 h, 12–14 h and 17–20 h; snack: other). ▪ Based on energy percentage contribution (meal: ≥ 15%, snack: < 15% of total energy).	DQ (HEI-2010).	Age group, race and ethnicity, years of education, family poverty income ratio, smoking, any recreational PA, weight status, dietary reporting status, and survey cycle.	Every additional eating occasions was positively associated to DQ in all measures (*p* < 0.01): Men: ▪ EF (β = 1.77). ▪ MF-%energy (β = 4.09), MF-self-report (β = 4.22) and MF-clock time (β = 2.14). ▪ SF-%energy (β = 1.52), SF-self-report (β = 1.25) and SF- clock time (β = 1.28). Women: ▪ EF (β = 2.22). ▪ MF-%energy (β = 3.62), MF-self-report (β = 5.35) and MF-clock time (β = 2.70). ▪ SF-%energy (β = 1.97), SF-self-report (β = 1.52) and SF- clock time (β = 1.57).
Leech 2016 [57].	▪ Australian. ▪ *n* = 4323 (2270 women and 2053 men). ▪ Age: ≥19 years.	Eating frequency: ▪ Eating occasion: food/beverages with ≥ 210 kJ (1–3, 4–5 or ≥6). ▪ Meals: breakfast, brunch, lunch, dinner, or supper (1–2 or ≥ 3). ▪ Snacks: 0–1, 2–3 or ≥ 4.	DQ (DGI-2013).	Age, education, income, country of birth, PA, total sedentary time, smoking, alcohol, currently dieting, eating more or less than usual, and ratio of reported total energy intake.	▪ Eating occasion: men (β = 1.38 [95% CI: 0.71, 2.05]) and women (β = 1.12 [95% CI: 0.34, 1.90]). ▪ MF: men (β = 5.60 [95% CI: 3.89, 7.34]) and for women (β = 4.11 [95% CI: 2.23, 5.93]).
House 2018 [63].	▪ American (Hispanic). ▪ *n* = 92 (47 women and 45 men). ▪ Age: 18–19 years.	Eating frequency: infrequent (<3 meals/day) or frequent (>4 meals/day).	BMI, BMI z-score, BW, WC.	Age, sex and percent time spent in moderate to vigorous physical activity.	Infrequent eaters vs. frequent eaters: ▪ Total population: > BMI (*p* = 0.02) and BMI z-score (*p* = 0.03). In stratified analyses by sex: ▪ Women: > BMI (*p* = 0.04). Other variables: NA.
Kim 2018 [23].	▪ South Korean. ▪ *n* = 6951 (3487 women and 3464 men). ▪ Age: 19–93 years.	▪ Eating frequency: all eating occasions (<3, 4 or ≥5). ▪ Meal frequency: 1, 2 or 3. ▪ Snack frequency: 0, 1, 2 or 3.	BMI, WC, DQ (MAR).	Age group, sex, smoking, alcohol drinking frequency, PA, resistance PA frequency, household income, education level, stress level, EI, depressed mood, meal frequency, and snack frequency.	In ≥ 5 EF vs. < 3 EF: ▪ <BMI (*p* < 0.01) and WC (*p* < 0.01). ▪ <BMI (*p* < 0.01) and WC (*p* < 0.01) in highest DQ. 3 MF vs. 2 MF:▪ <BMI (*p* < 0.04) and WC (*p* < 0.01). ▪ <WC (*p* < 0.01) in highest DQ. 0 SF vs. 2 SF: ▪ <BMI (*p* < 0.01) and WC (*p* < 0.01). ▪ <BMI (*p* < 0.01) in highest DQ.
Alamri 2020 [59].	▪ Saudi. ▪ *n* = 435 women. ▪ Age: 20–25 years.	Snack frequency: Other eating occasions besides breakfast, lunch, or dinner.	WC ≤ 88 cm, WC > 88 cm, BMI 18.5 to < 25 and BMI ≥ 25.	None.	▪ > SF in the evening: > frequent in WC> 88 cm (*p* = 0.04) and BMI≥ 25 (*p* = 0.04). ▪ Other variables: NA.

Abbreviations: AO, abdominal obesity (waist-height > 0.5); β, beta coefficient; BMI, body mass index; BP, blood pressure; BW, body weight; CASPIAN-IV, childhood and adolescence surveillance and prevention of adult non-communicable disease; DBP, diastolic blood pressure; DGI, dietary guidelines index; DQ, diet quality; DQS, dietary quality score; EF, eating frequency; ELFA, early life factors adjustment; HC, hip circumference; HDI, health diet indicator; HDL-c, high density lipoprotein cholesterol; HT, hypertension; INTERMAP, international study of macro-and micro-nutrients; IPAQS, international physical activity questionnaire score; LCFA, later childhood factors adjustment; MAR, mean adequacy ratio; MDS, Mediterranean diet score; MF, meal frequency; NA, no association; OB, obesity; OR, odds ratio; OW, overweight; PA, physical activity; r, PANIC, physical activity and nutrition in children; Pearson’s correlation; SBP, systolic blood pressure; SF, snack frequency; SOLAR, study of Latino adolescents at risk for diabetes; SPEEDY study; sport, physical activity and eating behavior: environmental determinants in young people; TG, triglycerides; vs., versus; WC, waist circumference.

**Table 3:** Characteristics and main findings of longitudinal studies that explored eating speed/rate.

Longitudinal Studies					
**Children**					
**Author and Year**	**Population**	**Exposure (s)**	**Outcome (s)**	**Adjusted Variables**	**Results**
Okubo 2017 [28].	▪ Osaka Maternal and Child Health Study. ▪ Japanese. ▪ *n* = 492 mother-child pairs. ▪ Age: 30–42 m. ▪ 1 year follow-up.	Eating rate (self-reported).	BMI.	Child’s sex and age (at fourth survey), maternal age and BMI at enrollment, education level, family income, pregnancy smoking status, maternal working status at 30 months postpartum, birth order, birth weight, duration of breast-feeding, time spent watching TV at 30 months of age, protein intake, fat intake, and dietary fiber at 30 months of age.	Fast eating rate vs. slow eating rate at 30 m.: ▪ BMI at 42 m. (β = 0.67 [95% CI: 0.24, 1.10]).
**Adults**					
**Author and Year**	**Population**	**Exposure (s)**	**Outcome (s)**	**Adjusted Variables**	**Results**
Tanihara 2011 [9].	▪ Japanese. ▪ *n* = 529 men. ▪ Age: 20–59 years. ▪ 8 year follow-up.	Eating speed (self-reported).	OW.	Age, drinking, smoking, regular exercise.	▪ Fast vs. medium or slow eating speed: OW (OR = 1.80 [95% CI: 1.25, 2.59]).
Yamane 2014 [2].	▪ Japanese. ▪ n = 1314 (638 women and 676 men). ▪ Pre-universities. ▪ 3 year follow-up.	Eating quickly.	OW.	Gender, eating quickly, frequently consuming fatty foods.	▪ Eating quickly vs. no eating quickly: OW (OR = 4.40 [95% CI: 2.22, 8.75]).
Zhu 2015 [5].	▪ Japanese ▪ *n* = 8941 (5517 women and 3424 men). ▪ Age: 40–75 years. ▪ 3 year follow-up.	Eating speed (self-reported).	MetS, WC, HDL-c, TG, BP and FPG.	Age and sex, drinking alcohol, dietary behavior, physical activity, sleeping, and Medication history.	▪ Fast eating speed vs. not fast eating speed: HR for MetS (HR = 1.30 [95% CI: 1.05, 1.60]), WC (HR = 1.35 [95% CI: 1.10, 1.66]) and lower HDL-c (HR = 1.37 [95% CI: 1.12, 1.67]). ▪ Other variables: NA.
Leong 2016 [56].	▪ New Zealander. ▪ *n* = 1014 women. ▪ Age: 40–50 years. ▪ 3 year follow-up.	Eating speed (self-reported).	OW.	Baseline BMI, age, socioeconomic status, thyroid condition, ethnicity, change in physical activity, change in smoking status and change in menopause status.	▪ No significant associations for OW.

Abbreviations: β, beta coefficient; BMI, body mass index; BP, blood pressure; FPG, fasting plasma glucose; HDL-c, high density cholesterol; HR, hazard ratio; M, months of age; MetS, metabolic syndrome; NA, no associations; OB, obesity; OR, odds ratio; OW, overweight; TG, triglycerides; vs., versus; WC, waist circumference.

**Table 4:** Characteristics and main findings of longitudinal studies that explored eating frequency.

Longitudinal Studies					
**Children**					
**Author and Year**	**Population**	**Exposure (s)**	**Outcome (s)**	**Adjusted Variables**	**Results**
Franko 2008 [31].	▪ American (black and white race). ▪ *n* = 2375 girls. ▪ Age: 9–10 years. ▪ 10 year follow-up.	Meal frequency: number of days consumed ≥ 3 meals (breakfast, snack, lunch or other).	BMI-for-age z-score and OW.	Visit, study site, parental education, socioeconomic status, race, energy intake and indicators of physical activity.	▪ MF: BMI-for-age z score (β = −0.047, *p* < 0.01).
Ritchie 2012 [30].	▪ NGHS study. ▪ American (black and white race). ▪ *n* = 2372 girls. ▪ Age: 9–10 years. 10 year follow-up.	Eating frequency: ▪ Eating episode: 1–3, 3.1–4, 4.1–6 or >6 per day. ▪ Meal: 1–2.5 or > 2.5 meals per day. ▪ Snack: 0–1, 1.1–2, 2.1–3 or > 3 per day.	BMI and WC.	BMI or WC, race, parental education, physical activity, Television/video viewing, and total energy intake, dieting for weight loss.	▪ >6 total eating episodes/day: <BMI (*p* = 0.01) and WC (*p* = 0.04). ▪ >2.5 MF: >BMI (*p* = 0.04).
Taylor 2017 [4].	▪ New Zealander. ▪ *n* = 371 (175 girls and 196 boys). ▪ Age: 1–3.5 years. ▪ 3.5 year follow-up	Eating frequency (all eating occasions).	BMI z-score.	POI intervention group, household factors, maternal parity/education, infant sex, birth weight, pre-pregnancy BMI, pregnancy smoking, and exclusive breastfeeding.	No significant associations.
**Adults**					
**Author and Year**	**Population**	**Exposure (s)**	**Outcome (s)**	**Adjusted Variables**	**Results**
Kahleova 2017 [67].	▪ AHS-2. ▪ North American. ▪ *n* = 50,660. ▪ Age: ≥ 30 years. ▪ 7 ± 1 year follow-up.	Meal frequency and timing: ▪ Breakfast: from 5–11 h. ▪ Lunch: from 12–16 h. ▪ Dinner: from 17–23 h.	BMI.	Age, sex, ethnicity, marital status, education, personal income, dietary pattern, exercise, sleep, television watching, energy intake, and high blood pressure medicine.	▪ ≥6 MF vs. 3 MF: BMI (β = 0.04 [95% CI: 0.02, 0.06]).
Larsen 2019 [74].	▪ MONICA study. ▪ Danish. ▪ *n* = 2124 (1044 women and 1080 men). ▪ Middle-aged. ▪ 6 year follow-up.	Total eating, meal and snack frequency (self-reported).	BMI and WC.	Baseline measure of outcome, smoking, alcohol, PA, education, age, gender, menopausal status for women, and height (in WC analysis only).	Baseline MF: ▪ 6-y. change: BMI (β = −0.14 [95% CI: −0.27, 0.00]) and WC (β = −0.49 [95% CI: −0.99, 0.00]).

Abbreviations: ADHS-2, Adventist Health Study 2; β, beta coefficient; BMI, body mass index; EF, eating frequency; MF, meal frequency; MONICA, Danish Monitoring Trends and determinants in Cardiovascular Disease; NA, no associations; NGHS, National Heart, Lung, and Blood Institute Growth and Health Study; OR, odds ratio; OW, overweight; PA, physical activity; POI, Prevention of Overweight in Infancy; vs., versus; WC, waist circumference.

**Table 5:** Characteristics and main findings of interventional trials that explored eating speed/rate.

Interventional Trials						
**Children**						
**Author and Year**	**Study Design**	**Population**	**Intervention Description**	**Comparing Group**	**Adjusted Variables**	**Results**
Faith 2019 [12].	Parallel: 8 weeks.	▪ American. ▪ *n* = 24 girls and boys. ▪ Age: 4–8 years.	RePace: ▪ 5 interactive sessions of 1 h for parents and children, over 8 weeks. ▪ Small timers were given at the clinic, programmed to vibrate at 30-s intervals during the mealtimes and snacks. ▪ Chat Jar during mealtimes to encourage a slower eating. DUC (at the end of the 8 weeks): ▪ 30 min of informative educational sessions (healthy eating recommendations, importance of family meals, slowly eating speed).	RePace or DUC.	Child age, sex and baseline BMI and BMI z-score.	▪ Intervention: BMI (β = −0.57, *p* = 0.02) and BMI z-score (β = −0.23, *p* = 0.03).

Abbreviations: β, beta coefficient; BMI, body mass index; DUC, delayed usual care; RePace, reduced eating pace.

**Table 6:** Characteristics and main findings of interventional trials that explored eating frequency.

Interventional Trials						
**Adults**						
**Author and Year**	**Study Design**	**Population**	**Intervention Description**	**Comparing Group**	**Adjusted Variables**	**Results**
Carlson 2007 [70].	Crossover: 18 weeks.	▪ American. ▪ *n* = 15 (10 women and 5 men). ▪ Age: 40–50 years.	▪ Controlled diet: 3 meals/day: breakfast, lunch and dinner or 1 meal/day: during 4 Hours in the early evening (16–20 h). ▪ 11-weeks off-diet.	3 MF or 1 MF/day.	Period-specific baseline values.	▪ 3 MF vs. 1 MF: < FPG (*p* < 0.01).
Stote 2007 [72].	Crossover: two 8 weeks periods.	▪ American. ▪ *n* = 15 (10 women and 5 men). ▪ Age: 40–50 years.	1 meal/day: during a 4 h period in the early evening.	3 MF/day (breakfast, lunch and dinner).	First observation within a period.	3 MF vs. 1 MF: ▪ < SBP (*p* = 0.02) and DBP (*p* = 0.04). ▪ > BW (*p* = 0.01). 3 MF vs. 1 MF/day: ▪ < HLD-c (*p* = 0.01). ▪ FPG and TG: NA.
Kanaley 2014 [68].	Crossover: 3 days with a 12 h period each one.	▪ American. ▪ *n* = 14 (11 women and 3 men). ▪ Age: 20–59 years.	▪ Low frequency (3MF), high frequency (6MF) or high frequency + high protein (6MFHP). ▪ Wash out: 1 month between each study day.	Low frequency, high frequency or high frequency + protein regimen.	None.	▪ 6 MFHP vs. 3 MF or 6 MF: < FPG tAUC (*p* < 0.01). ▪ 3 MF vs. 6 MF: NA for FPG.
Alencar 2015 [69].	Crossover: 6-weeks.	▪ American. ▪ *n* = 11 women. ▪ Age: 35–60 years.	2 MF pattern (every 5–6 h), 6 MF pattern: (every 2–3 h) or washout phase (4 MF).	2 MF pattern, 6 MF pattern or washout phase.	None.	▪ 2 MF vs. 6 MF: > HDL-c (*p* < 0.05).
Megson 2017 [71].	Parallel: 3 months.	▪ American. ▪ *n* = 211 (176 women and 35 men). ▪ Age: 18–70 years.	▪ Group SURI program: website + a pedometer + periodic newsletters; community exercise programs + prizes and recognition. ▪ Group SURI + IBWL: SURI program + internet Behavioral weight loss program. ▪ Group SURI + IBWL + Group: included Weekly group meetings.	SURI program, SURI + IBWL and SURI + IBWL + Group.	Treatment arm.	▪ EF: NA for BW loss.
Yildiran 2019 [73].	Parallel: 3 months.	▪ Turkish. ▪ *n* = 47 women. ▪ Age: 20–49 years.	3 meals/day (3 main meals) or 6 meals/day (3 main meals + 3 snacks).	3 MF or 6 MF/day.	None.	▪ Not significant effects in BMI, WC, BW, FPG, TG, HDL-c between groups.

Abbreviations: BMI, body mass index; BW, body weight; DBP, diastolic blood pressure; EF, eating frequency; FPG, fasting plasma glucose; HDL-c, high density lipoprotein cholesterol; HMF, high meal frequency; IBWL, Internet behavioral weight loss program; LMF, low meal frequency; MF, meal frequency; NA, no association; NE, no effect; PPG, postprandial peak of glucose; SBP, systolic blood pressure; SURI, Shape Up Rhode Island; tAUC, total area under the curve; TG, triglycerides; vs., versus; WC, waist circumference.
